# Tricuspid annular plane systolic excursion and central venous pressure in mechanically ventilated critically ill patients

**DOI:** 10.1186/s12947-018-0130-2

**Published:** 2018-08-07

**Authors:** Hongmin Zhang, Xiaoting Wang, Xiukai Chen, Qing Zhang, Dawei Liu

**Affiliations:** 1 Department of Critical Care Medicine, Peking Union Medical College Hospital, Chinese Academy of Medical Sciences, 1# Shuai Fu Yuan, Dong Cheng District, Beijing, 100730 China; 2 Pittsburgh Heart, Lung, Blood and Vascular Medicine Institute, University of Pittsburgh, Pittsburg, PA 15261 USA

**Keywords:** Echocardiography, Tricuspid annulus plane systolic excursion, Central venous pressure, Critically ill

## Abstract

**Background:**

The tricuspid annular plane systolic excursion (TAPSE) is commonly recommended for estimating the right ventricular systolic function. The central venous pressure (CVP), which is determined by venous return and right heart function, was found to be associated with right ventricular outflow fractional shortening. This study thus aimed to investigate the relationship between the TAPSE and CVP in mechanically ventilated critically ill patients.

**Methods:**

This is a prospective observational study. From October 1 to December 31, 2017, patients admitted to the intensive care unit with CVP monitoring and controlled mechanical ventilation were screened for enrolment. Echocardiographic parameters, including the TAPSE, mitral annular plane systolic excursion (MAPSE), left ventricular ejection fraction (LVEF), and internal diameter of inferior vena cava (dIVC), and haemodynamic parameters, including the CVP, were collected.

**Results:**

Seventy-four patients were included. Thirty-one were included in the low LVEF (< 55%) group, and 43 were included in the high LVEF (≥55%) group. In the high LVEF group, the TAPSE and CVP were not correlated (*r* = − 0.234, *P* = 0.151). In the low LVEF group, partial correlation analysis indicated that the TAPSE and CVP were correlated (*r* = − 0.516, *P* = 0.006), and multivariable linear regression analysis indicated that the TAPSE was independently associated with the CVP (standard coefficient: − 0.601, *p* < 0.001). Additionally, in the low LVEF group, a ROC analysis showed that the area under the curve of the TAPSE for the detection of CVP greater than 8 mmHg was 0.860 (95% confidence interval: 0.730–0.991; *P* = 0.001). The optimum cut-off value was 1.52 cm, which resulted in a sensitivity of 75.0%, a specificity of 86.7%, a positive predictive value of 84.6% and a negative predictive value of 77.8%.

**Conclusions:**

The TAPSE is inversely correlated with the CVP in mechanically ventilated critically ill patients who have a LVEF less than 55%.

## Background

Echocardiography is a noninvasive diagnostic tool and can provide important information regarding certain haemodynamic parameters [1]. Among the measures of the right ventricle (RV) systolic function, the tricuspid annular plane systolic excursion (TAPSE) is easily applied and has low inter-observer variability [2, 3]. The American Society of Echocardiography recommend using the TAPSE routinely as a simple method to estimate the RV systolic function [4]. The TAPSE has also been shown to have prognostic value both in patients with pulmonary hypertension and heart failure and in noncardiac critically ill patients [5–7].

The Central venous pressure (CVP) is widely recognized as a useful parameter for managing critically ill patients. The CVP is determined by venous return and RV function and plays an important role in the monitoring and management of right ventricular failure patients [8–11]. Even though the CVP can only provide information about fluid responsiveness in extreme values, it is still useful when it is followed over time [12, 13]. Furthermore, the CVP can be used as a safety limit to avoid extra thoracic organ oedema because the risks for peripheral oedema, renal impairment and liver impairment are related to the absolute CVP value [14, 15]. Additionally, the CVP is of great value in the prognosis of critically ill patients [16, 17].

A previous study found that right ventricular outflow fractional shortening, which is used to assess right ventricular systolic function, can be used to predict the central venous pressure [18]. In a large cohort of healthy subjects, Ferrara F et al. noted that the TAPSE was correlated with echo-Doppler indices reflecting preload [19]. However, the relationship between the CVP and TAPSE has rarely been reported in the critically ill. The present study aimed to investigate the relationship between the TAPSE and CVP in mechanically ventilated critically ill patients.

## Methods

### Study population

Consecutive patients admitted to Peking Union Medical College Hospital Intensive Care Unit (ICU) from October 1 to December 31, 2017, were screened for enrolment within the first 24 h of being admitted.

Patients were included if they had central venous pressure monitoring and mechanically ventilation without spontaneous breath effort.

The exclusion criteria were post-cardiac surgery, acute Cor Pulmonale or severe pulmonary arterial hypertension (PAH), severe valvular disease, dilated or hypertrophic cardiomyopathy, constrictive pericarditis, Takotsubo syndrome, acute myocardial infarction, a non-sinus rhythm, intra-abdominal hypertension and an inadequate echocardiographic image for measurement.

This study was conducted according to the Declaration of Helsinki and was approved by the ethics committee of our institution. Written informed consent was obtained from the next of kin of each patient because all of the patients were in a state of unconscious.

### Echocardiography

Echocardiograms were performed using an echocardiograph (CX50, PHILIPS, USA) with a 2.5-MHz phased-array probe within the first 24 h of ICU admission. ECG was recorded continuously during the echo examination. Three cardiac cycles were analysed and averaged. The patients were in the semi left lateral position during the examination. Echocardiographic M-mode and Doppler measurements were taken in a standard manner. The images were recorded for offline analysis. Two intensivists with experience in echocardiography performed the examination.

**Fig. 1:**
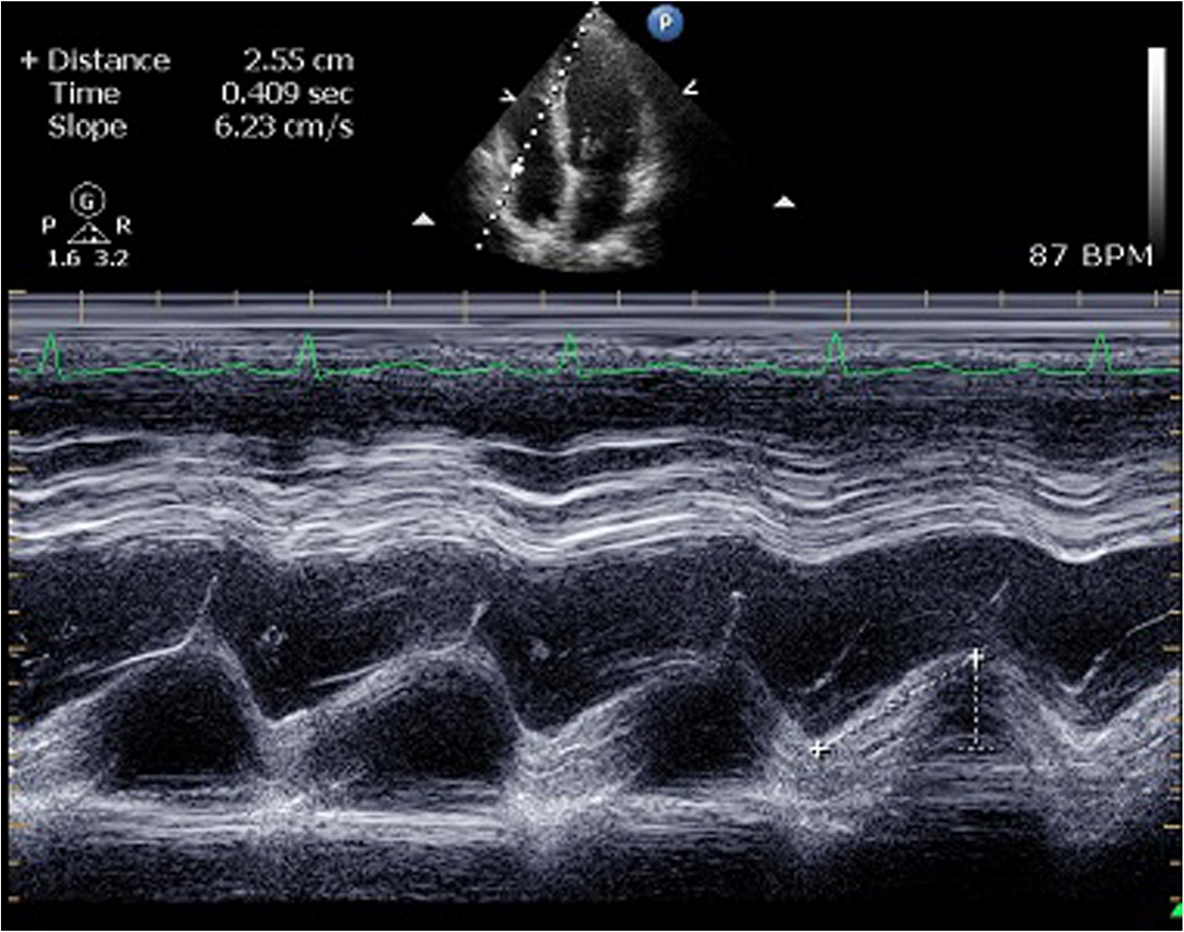
The measurement of TAPSE

The left ventricular ejection fraction (LVEF) was obtained using a modified biplane Simpson’s method from the apical two- and four-chamber views. Indexes of longitudinal systolic function measurements were taken from the apical four-chamber view. The mitral annular plane systolic excursion (MAPSE) was obtained by putting the cursor along the mitral ring and measuring the difference between the highest and lowest points of the M-mode sinusoid wave. The tricuspid annular plane systolic excursion (TAPSE) was obtained by putting the M-mode cursor along the lateral part of the tricuspid valve ring (Fig. 1). The ratio of end diastolic area of the right ventricle and left ventricle (R/LVEDA) was measured at an apical 4-chamber view during end-diastole. The left ventricular outflow tract (LVOT) velocity-time integral (VTI) was obtained from pulsed Doppler by putting the sample volume at the LVOT approximately 0.5 cm below the aortic valve [20]. The IVC was examined subcostally in the longitudinal view, and its diameter was measured at the end of expiration just upstream of the origin of the suprahepatic vein.

### Other parameters collected

The demographics, Acute Physiology and Chronic Health Evaluation (APACHE) II score, Sequential Organ Failure Assessment (SOFA) score, reason for admission and comorbidities were collected for each patient. A central line was placed in the internal jugular vein for all patients to allow for the CVP measurement. We recorded the heart rate (HR), mean arterial pressure (MAP), CVP, vasoactive agents and ventilator settings at the onset of the echo examination.

### Statistical analysis

The statistical analysis was performed using the SPSS 13.0 statistical software package (SPSS Inc., Chicago, Illinois, USA). Continuous data were expressed as the mean ± SD or as the median and the interquartile range. Categorical variables were presented as the number and the percentages. Normal distribution of the continuous values was assessed by the Kolmogorov-Smirnov test. Group comparisons were performed by Students’ t test, Mann-Whitney U test or Chi-squared test or Fisher’s exact test where appropriate. A partial correlation test was used to assess univariate relations. Multivariable linear regression analysis, including all echocardiographic parameters from the univariate analysis, was constructed to assess the independent associations of these variables with the CVP. Receiver-operating characteristic (ROC) curves were analysed, and the areas under each respective curve were calculated and compared. All *p*-values were two tailed and were considered significant when *p* < 0.05. Intraobserver and interobserver variability on TAPSE, dIVC, LVEF were assessed in 20 randomly selected patients and were tested using both paired t tests and intraclass correlation coefficients (ICCs). An ICC ˃0.8 was considered excellent agreement.

## Results

### General characteristics of all patients

**Fig. 2:**
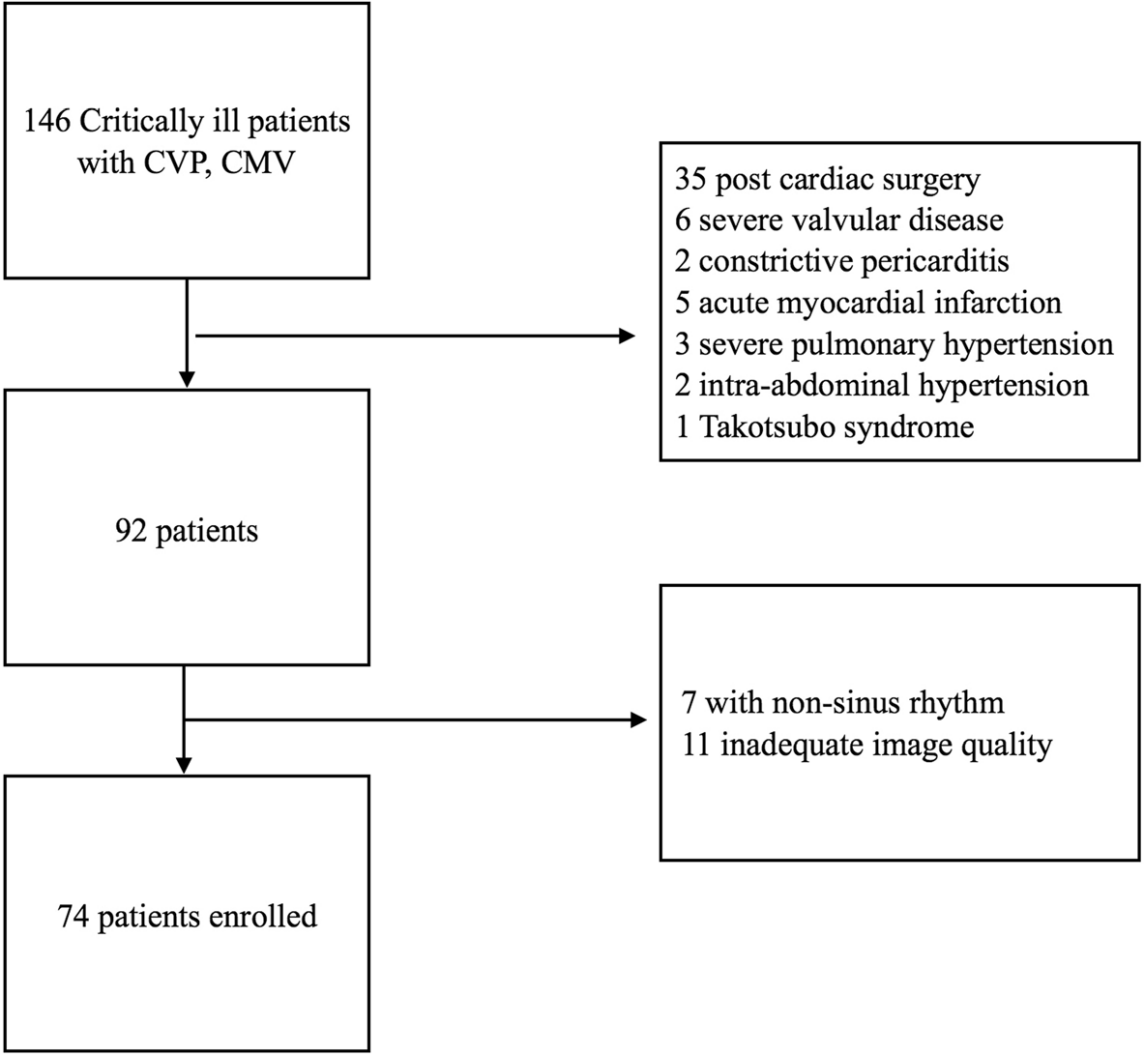
Flow chart of patient enrollment

A total of 146 patients were screened for enrolment in the study and 74 were included; of them, 31 patients were placed in the low LVEF (< 55%) group and the remaining 43 were placed in the high LVEF (≥55%) group (Fig. 2). The general characteristics of the patients are illustrated in Table 1. The reasons for admission to ICU included sepsis, high risk surgery and others (stoke, renal failure, and severe electrolyte disturbances). No difference was found between the two groups in terms of age, sex, reason for admission and comorbidities. The low LVEF group had higherAPACHE II and SOFA scores, 20.6 vs 16.9, *p* = 0.032 and 9.5 vs 7.4, *p* = 0.005, respectively. The low LVEF group had more patients being administered norepinephrine (NE) and had a larger dose, 71.9% vs 46.5%, *p* = 0.027 and 0.35 μg/kg/min vs 0.18 μg/kg/min, *p* = 0.008, respectively. No differences were found in the proportion of ARDS patients, positive end expiration pressure (PEEP) and plateau pressure (Pplat) between the two groups.

**Table 1:** General Characteristics

Categories	LVEF<55%(*n* = 31)	LVEF≥55%(*n* = 43)	*p*
Age (yr)	67.4 ± 16.5	61.1 ± 15.5	0.096
Sex (male, %)	23 (71.9%)	26 (60.5%)	0.303
APACHEII	20.6 ± 8.1	16.9 ± 6.2	0.032
SOFA	9.5 ± 2.6	7.4 ± 3.0	0.005
Reason for admission (n, %)			
Sepsis	18 (56.3%)	22(51.2%)	0.674
High-risk Surgery	10 (31.3%)	19(44.2%)	0.250
Others	4 (12.5%)	2 (4.7%)	0.220
Comorbidities (n, %)			
HTN	13 (40.6%)	14 (32.6%)	0.466
CAD	4 (12.5%)	4 (9.3%)	0.650
DM	6 (18.7%)	7 (16.3%)	0.762
Stroke	2 (6.3%)	1 (2.3%)	0.405
NE (n, %)	23 (71.9%)	20 (46.5%)	0.027
NE dose (μg/kg/min)	0.35 (0.19, 0.60)	0.18 (0.06, 0.30)	0.008
ARDS (n, %)	7 (22.6%)	5 (11.6%)	0.201
PEEP (mmHg)	6.1 ± 1.9	5.7 ± 1.4	0.310
Pplat (mmHg)	16.1 ± 4.7	15.9 ± 2.9	0.798

*Others: stroke, renal failure, severe electrolyte disturbances

*APACHE* acute physiology and chronic health evaluation, *SOFA* sequential organ failure assessment, *HTN* hypertension, *CAD* coronary arterial disease, *DM* diabetes mellitus, *NE* norepinephrine, *ARDS* acute respiratory distress syndrome, *PEEP* positive end expiratory pressure, *Pplat* plateau pressure

### Haemodynamic and echocardiographic parameters

The mean LVEFs in the low LVEF group and high LVEF group were 45 and 69%, respectively. The low LVEF group had a higher CVP than the high LVEF group did, but this difference was not statistically significant (9 mmHg vs 8 mmHg, *p* = 0.056). Compared with the high LVEF group, the low LVEF group had a significantly lower VTI, 16.3 cm vs 20.9 cm, *p* < 0.001. The low LVEF group also had a significantly lower TASPE and MAPSE, 1.61 cm vs 2.15 cm, p < 0.001 and 1.17 cm vs 1.55 cm, p < 0.001, respectively. The low LVEF group had a greater dIVC, 1.8 cm vs 1.6 cm, *p* = 0.017. None of the patients in the two groups were found to have a R/LVEDA>1, and the proportion of patients with a R/LVEDA 0.6–1 and R/LVEDA < 0.6 between the groups was not significantly different. No difference was found in the HR and MAP between the groups (Table 2).

**Table 2:** Hemodynamics and echocardiographic parameters

Categories	LVEF<55%(*n* = 31)	LVEF≥55%(*n* = 43)	*p*
HR (bpm)	95 ± 19	90 ± 21	0.351
MAP (mmHg)	87 ± 15	91 ± 16	0.240
CVP (mmHg)	9 (8, 10)	8 (5, 10)	0.056
R/LVEDA (n, %)			
>1	0	0	–
0.6–1	20 (64.5%)	23(53.5%)	0.351
<0.6	11 (35.5%)	20 (46.5%)	0.351
TAPSE (cm)	1.61 ± 0.49	2.15 ± 0.37	<0.001
MAPSE (cm)	1.17 ± 0.42	1.55 ± 0.37	<0.001
dIVC (cm)	1.8 ± 0.3	1.6 ± 0.4	0.017
VTI (cm)	16.3 ± 4.5	20.9 ± 5.7	<0.001
LVEF (%)	45 ± 9	69 ± 6	<0.001

*HR* heart rate, *MAP* mean arterial pressure, *CVP* central venous pressure, *R/LVEDA* ratio of end diastolic area between right and left ventricle, *TAPSE* tricuspid annular plane systolic excursion, *MAPSE* mitral annular plane systolic excursion, *dIVC* internal diameter of inferior vena cava, *VTI* velocity-time integral, *LVEF* left ventricular ejection fraction

### Correlation between the CVP and echocardiographic parameters in the high LVEF group

In the univariate analysis, the CVP was positively correlated with the dIVC (*r* = 0.414, 95% confidence interval [CI]: 0.061–0.680, *p* = 0.009). No significant correlation was found between the CVP and other parameters, including the TAPSE, MAPSE, VTI and LVEF. In a multivariable analysis, the dIVC was the only independent variable associated with the CVP (standard coefficient 0.522, *p* < 0.001) (Table 3).

**Table 3:** Significant independent relation of CVP with echocardiographic variables in high LVEF group

Variables	Univariate analysis			Multivariate analysis	
		95%CI	*P*	Std coefficient (β)	*P*
TAPSE	−0.234	−0.516 to 0.161	0.151	−0.215	0.107
dIVC	0.414	0.061 to 0.680	0.009	0.522	<0.001
MAPSE	− 0.014	−0.292 to 0.287	0.932	− 0.134	0.347
VTI	0.283	0.017 to 0.516	0.081	0.230	0.118
LVEF	0.133	−0.200 to 0.475	0.420	0.107	0.430

*TAPSE* tricuspid annular plane systolic excursion, *MAPSE* mitral annular plane systolic excursion, *dIVC* internal diameter of inferior vena cava, *VTI* velocity-time integral, *LVEF* left ventricular ejection fraction, *Std* standard

### Correlation between the CVP and echocardiographic parameters in the low LVEF group

**Fig. 3:**
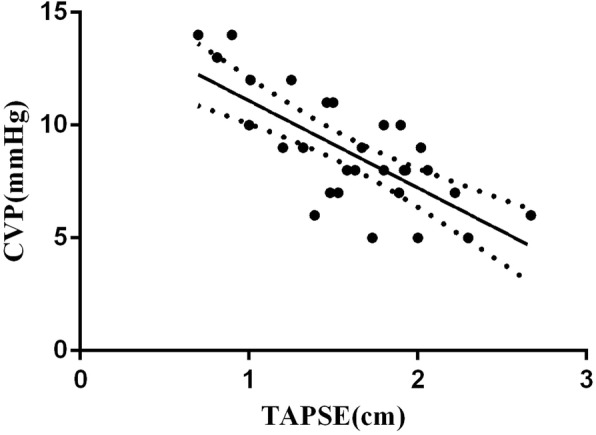
Correlation between TAPSE and CVP in patients with LVEF below 55%. CVP was negatively correlated with TAPSE, *r* = − 0.516, *P* = 0.006

In the univariate analysis, the CVP was positively correlated with the dIVC (*r* = 0.390, 95%CI: 0.012 to 0.689, *p* = 0.044) and negatively correlated with the TAPSE (*r* = − 0.516, 95%CI: − 0.132 to − 0.799, *P* = 0.006) (Fig. 3). No significant correlation was observed between the CVP and other parameters, including the MAPSE, VTI and LVEF. In a multivariable analysis, the TAPSE and dIVC were independent variables associated with the CVP (standard coefficient − 0.601, *p* < 0.001 and standard coefficient 0.300, *p* = 0.030, respectively) (Table 4).

**Table 4:** Significant independent relation of CVP with echocardiographic variables in low LVEF group

Variables	Univariate analysis			Multivariate analysis	
	r	95%CI	*P*	Std coefficient (β)	*P*
TAPSE	− 0.516	− 0.132 to − 0.799	0.006	− 0.601	<0.001
dIVC	0.390	0.012 to 0.689	0.044	0.300	0.030
MAPSE	0.021	−0.447 to 0.546	0.918	0.004	0.978
VTI	−0.239	−0.489 to 0.018	0.231	−0.170	0.763
LVEF	−0.067	−0.455 to 0.274	0.741	−0.029	0.886

*TAPSE* tricuspid annular plane systolic excursion, *MAPSE* mitral annular plane systolic excursion *dIVC* internal diameter of inferior vena cava, *VTI* velocity-time integral, *LVEF* left ventricular ejection fraction, *Std* standard

**Fig. 4:**
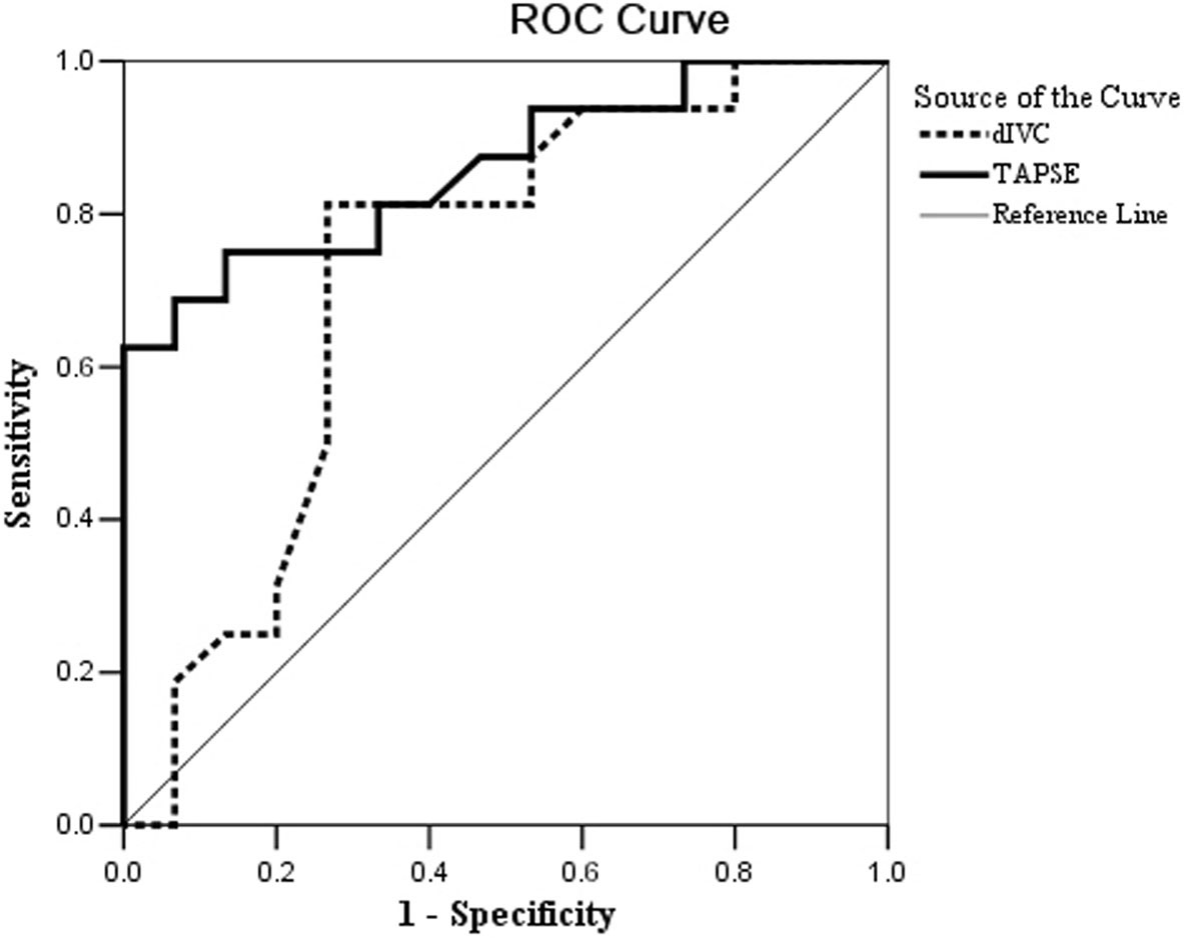
ROC curve to detect CVP greater than 8 mmHg in patients with LVEF below 55%. The area under the curve for TAPSE to detect CVP >8 mmHg in patients with LVEF <55% is 0.860 (95%CI 0.730, 0.991), *P* = 0.001, TAPSE at 1.52, sensitivity 75.0%, specificity 86.7%. The area under the curve for dIVC to detect CVP >8 mmHg in patients with LVEF <55% is 0.723 (95%CI 0.533, 0.913), *P* = 0.034, dIVC at 1.8, sensitivity 68.8%, specificity73.3%. Area comparison, Z = − 1.162, *P* = 0.245

To evaluate the sensitivity and specificity of the two parameters for detecting a CVP greater than 8 mmHg, ROC curves were calculated (Fig. 4). The ROC analysis showed that the TAPSE was a good marker, with an area under the curve (AUC) of 0.860 (95% CI: 0.730–0.991, *P* = 0.001). The AUC of the dIVC was 0.723 (95%CI: 0.533–0.913, *p* = 0.034). The two AUCs were not statistically different (Z = − 1.162, *p* = 0.245).

For the TAPSE, the optimum cut-off value was 1.52 cm, which resulted in a sensitivity of 75.0%, a specificity of 86.7%, a positive predictive value (PPV) of 84.6% and a negative predictive value (NPV) of 77.8%. For the dIVC, the optimum cut-off value was 1.8 cm, which resulted in a sensitivity of 68.8%, a specificity of 73.3%, a PPV of 64.2% and an NPV of 76.5%.

### Measurement variability

The intraobserver variabilities on TAPSE, dIVC and LVEF were minimal. The interobserver variability analysis revealed that ICCS regarding TAPSE, dIVC and LVEF were respectively: 0.937 (95%CI:0.824–0.978), 0.987 (95%CI:0.948–0.997) and 0.925 (95%CI:0.792–0.974).

## Discussion

TAPSE is a clinically feasible parameter of the RV and has been proven to be a valuable prognostic marker in various cardiac diseases [5, 21, 22]. Interestingly, in this study, we observed striking differences on the relationship between the CVP and TASPE in patients with a LVEF below 55% and in those with a LVEF of 55% and greater. We observed that the TAPSE was inversely correlated with the CVP in critically ill patients who had a LVEF below 55%. When adjusting for other possible confounding factors, the TAPSE remained an independent predictor of the CVP, and it was proven to be a good marker for discriminating whether the CVP was greater than 8 mmHg.

Volume is among the first steps of shock therapy [23]. Although dynamic parameters of volume responsiveness are recommended, they have their own innate weaknesses. Pulse pressure variation or stroke volume variation can only be used reliably in a subset of patients, i.e., those who are mechanically ventilated, sedated and without arrhythmias [24]. The passive leg raising test has fewer limitations but is not as simple to perform as it may seem at first glance; it also requires a close monitoring of stroke volume [25]. As a parameter, the CVP is far from perfect but is of great value in fluid therapy of the critically ill [26]. Our results suggest that TAPSE has the potential to predict the CVP in low LVEF patients and provides a noninvasive way to assess the right atrial pressure. The reliability of IVC respiratory variation to assess volume responsiveness has long been debated. No decisive conclusions on the accuracy of IVC respiratory variation can be drawn [27–29]. In consistency with prior studies, we found that dIVC is directly correlated with the CVP [30, 31]. However, dIVC has its own limitations. In a group of cardiac surgical patients, Lorsomradee S, et al. noted that the correlation between the dIVC and CVP was poor when the CVP was greater than 11 mmHg [32]. Other researchers also noted that the IVC dimension and collapsibility have limited utility in identifying the magnitude of CVP elevation [33, 34]. Thus, the TAPSE could be an alternative parameter in assessing right atrial pressure for low LVEF patients.

RV function, which is often compromised when facing an elevated afterload, could lead to elevation of the CVP. A linear inverse relationship was observed between the TAPSE and pulmonary vascular resistance in a group of PAH patients who had a mean systolic pulmonary pressure of 75 mmHg [5]. However, no such relationship was found among healthy subjects [19]. In this study, patients with severe PAH were excluded. No difference was found between the groups regarding the proportion of ARDS patients and the levels of PEEP and Pplat. We did include the measurement of the R/LVEDA and no difference was found between high and low LVEF patients. These results suggest that the two groups had the same risk for RV afterload elevation. Left ventricle (LV) dysfunction could also precipitate pulmonary arterial pressure through an elevated left atrial pressure [35]. However, in a group of heart failure patients, no relation was discovered between the TAPSE and maximal tricuspid regurgitation pressure gradient [22]. Therefore, the correlation of the TAPSE and CVP cannot be explained by RV afterload elevation in these low LVEF patients.

To date, the accepted definition of septic myocardiopathy is based on a depressed LVEF, but several studies reported that the RV function was also compromised [36, 37]. However, a significant part of the RV systolic function depends on the LV systolic function, and its incidence is difficult to identify [38]. The right ventricle is bounded by its free wall with transverse fibre orientation in the septum; this is essential for ventricular twisting, which is the vital mechanism for RV ejection [39]. An experimental study demonstrated that 30% of the contraction force of the RV comes from the LV [40]. A previous study reported that the TAPSE is reduced with LV dysfunction in heart failure patients, particularly with reduced septal longitudinal motion [41]. We speculated that the TAPSE was determined, to a greater degree, by the right ventricular function in patients with a low LVEF. Therefore, it is likely that TAPSE can reflect the RV function and volume load more precisely when treating patients with low LVEF. Although the mechanism needs further study to confirm it, the result of this study indicated that monitoring of the TAPSE in these patients holds greater value.

There are several limitations to this study. First, TAPSE is a load- and angle-dependent parameter that is reflective of both the right and left ventricle function. Furthermore, this is a single-centre study, and the sample size is insufficient to provide a definite conclusion. Nevertheless, it represents a useful pilot study for further prospective investigations with larger numbers of patients. Second, pulmonary arterial pressure was not measured directly through techniques like Swan-Ganz catheter, and only a few patients were found with measurable tricuspid regurgitation on echocardiography. This may limit the direct interpretation of RV afterload. Third, the subjects in this study were heterogeneous, and the cause of LV dysfunction was not addressed. Previous studies demonstrated that LV dysfunction was very common in ICU patients and that a variety of conditions could result in LV dysfunction, including severe sepsis, prolonged hypoxia, severe metabolic and multiorgan insults, and even tachyarrhythmias [42, 43]. Chockalingam A et al. noted that, to present a unified management approach, acute left ventricular dysfunction could be classified into global LV dysfunction, acute coronary syndrome, stress cardiomyopathy and myocardial injury with minor troponin elevations [42]. The present study excluded patients with severe valvular disease, severe pulmonary hypertension, acute myocardial infarction and Takotsubo syndrome. Therefore, only patients with normal LV function or patients with global LV dysfunction were enrolled. Moreover, the patients in this study reflected the makeup of the population referred for critical care in clinical practice.

## Conclusion

TAPSE is inversely correlated with CVP in mechanically ventilated critically ill patients who have a LVEF less than 55%.

## Abbreviations

CVP: Central venous pressure
TAPSE: Tricuspid annular plane systolic excursion
MAPSE: Mitral annular plane systolic excursion
dIVC: Internal diameter of inferior vena cava
LVEF: Left ventricular ejection fraction
RV: Right ventricle
ICU: Intensive care unit
PAH: Pulmonary arterial hypertension
R/LVEDA: Ratio of end diastolic area of the right ventricle and left ventricle
LVOT: Left ventricular outflow tract
VTI: Velocity-time integral
APACHE Π score: Acute Physiology and Chronic Health Evaluation Π score
SOFA: Sequential Organ Failure Assessment
HR: Heart rate
MAP: Mean arterial pressure
NE: Norepinephrine
PEEP: Positive end expiration pressure
Pplat: Plateau pressure
LV: Left ventricle
